# A rare case report of the successful withdrawal of a stent balloon that failed to deflate

**DOI:** 10.1186/s12872-023-03215-w

**Published:** 2023-04-11

**Authors:** Yong Yang, Shijun Yang, Xiang Cheng, Kun Liu

**Affiliations:** 1 Department of Cardiology, Union Hospital, Tongji Medical College, Huazhong University of Science and Technology, Wuhan, 430022 China; 2 Hubei Key Laboratory of Biological Targeted Therapy, Union Hospital, Tongji Medical College, Huazhong University of Science and Technology, Wuhan, 430022 China; 3 Hubei Provincial Engineering Research Center of Immunological Diagnosis and Therapy for Cardiovascular Diseases, Union Hospital, Tongji Medical College, Huazhong University of Science and Technology, Wuhan, 430022 China

**Keywords:** Percutaneous coronary intervention, Balloon deflation, Coronary artery, One-way valve, Case report

## Abstract

**Background:**

In current percutaneous coronary intervention (PCI) practice, the balloon used in the procedure should be deflated a short time after balloon dilation to avoid having prolonged balloon dilation in the coronary artery, which would block the coronary artery and cause myocardial ischemia. It is very rare for a dilated stent balloon to fail to deflate.

**Case summary:**

A 44-year-old male was admitted to the hospital due to chest pain after exercise. Coronary angiography showed severe proximal stenosis of the right coronary artery (RCA) consistent with a diagnosis of coronary artery disease, and coronary stent implantation was required. After the last stent balloon was dilated, the stent balloon could not be deflated and continued to expand, resulting in blockage of the RCA blood flow. The patient then suffered decreased blood pressure and heart rate. Finally, the stent balloon in its expanded state was forcefully and directly withdrawn from the RCA and successfully removed from the body.

**Conclusion:**

Deflation failure of a stent balloon is an extremely rare complication of PCI. Various treatment strategies can be considered based on hemodynamic status. In the case described herein, the balloon was pulled out of the RCA directly to restore blood flow, which kept the patient safe.

**Supplementary Information:**

The online version contains supplementary material available at 10.1186/s12872-023-03215-w.

## Background

Coronary artery disease (CAD) is a major burden for health care systems and economies worldwide [1]. Percutaneous coronary intervention (PCI) is a common approach in CAD to reduce the incidence of myocardial infarction (MI) and to reduce mortality rates in acute coronary syndrome [2]. PCI originated in the 1980s and has become increasingly popular [3]. Currently, more than 500,000 PCI procedures are performed annually worldwide [4]. Although many stent implantation operations are carried out successfully every day, the occurrence of a rare but potentially fatal situation during PCI should always be kept in mind [5].

## Case presentation

**Fig. 1:**
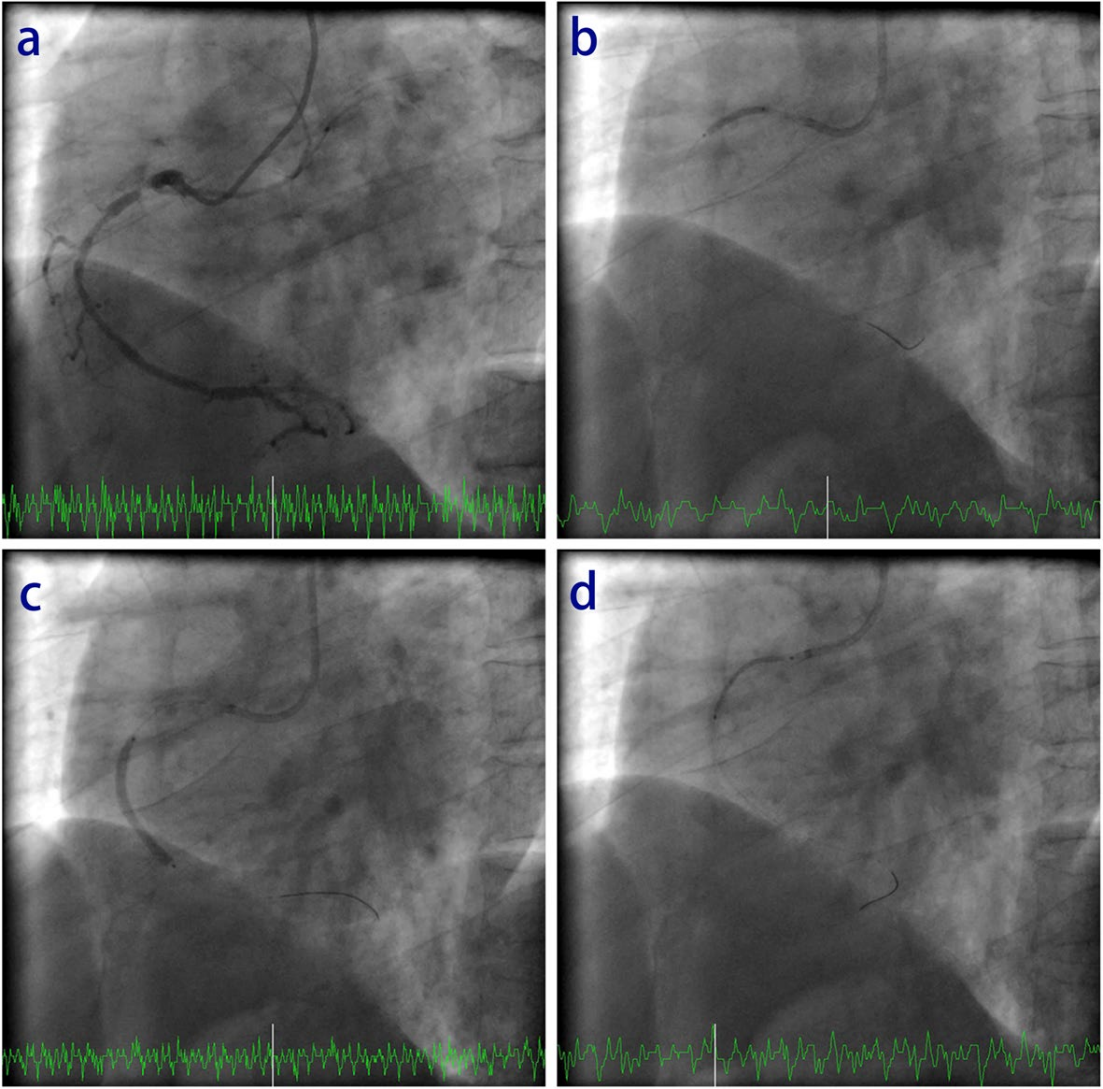
Coronary angiography and stent implantation in the RCA. **a** Coronary angiography showed diffuse arteriosclerosis with severe stenosis in the middle RCA, and the proximal RCA had 90% stenosis by diameter. **b** The stenotic region was dilated with a 2.0 mm*20 mm compliance balloon. **c** A 3.0 mm*33 mm drug-eluting stent (DES) was implanted in the middle of the RCA. **d** A 4.0 mm*33 mm drug-eluting stent was implanted in the proximal RCA

A 44-year-old male was admitted to the hospital due to chest pain after exercise and positive of the exercise treadmill test. His medical history was limited to moderate hypertension without medication. The patient had no past surgical/interventional history. After the patient was admitted, the electrocardiogram (ECG) and echocardiogram showed no obvious abnormalities. High-sensitivity troponin I (hsTnI) was normal. Blood lipid tests indicated moderate hyperlipidemia. A coronary angiography showed a balanced coronary dominant circulation, with diffuse and severe stenosis in the middle right coronary artery (RCA) and 90% stenosis by diameter in the proximal RCA (Fig. 1a, Supplementary Video 1), which was consistent with a diagnosis of CAD. Due to 90% stenosis in the RCA and typical ischemic symptoms with chest pain after exercise, as well as positive of the exercise treadmill test, PCI was indicated [6, 7]. With the consent of the patient and his family, PCI in the RCA was performed. Heparin (100 U/kg) was used during PCI. A 6Fr SAL 0.75 guiding catheter and SION-BLUE wire (Asahi, Tokyo, Japan) were used. First, the stenotic area was dilated with a 2.0 mm*20 mm compliance balloon (Fig. 1b, Supplementary Video 2). Then, a 3.0 mm*33 mm drug-eluting stent (Firebird, MicroPort, China) was implanted in the middle of the RCA (Fig. 1c, Supplementary Video 3). Finally, a 4.0 mm*33 mm drug-eluting stent (Firebird, MicroPort, China) was implanted in the proximal RCA (Fig. 1d, Supplementary Video 4).

**Fig. 2:**
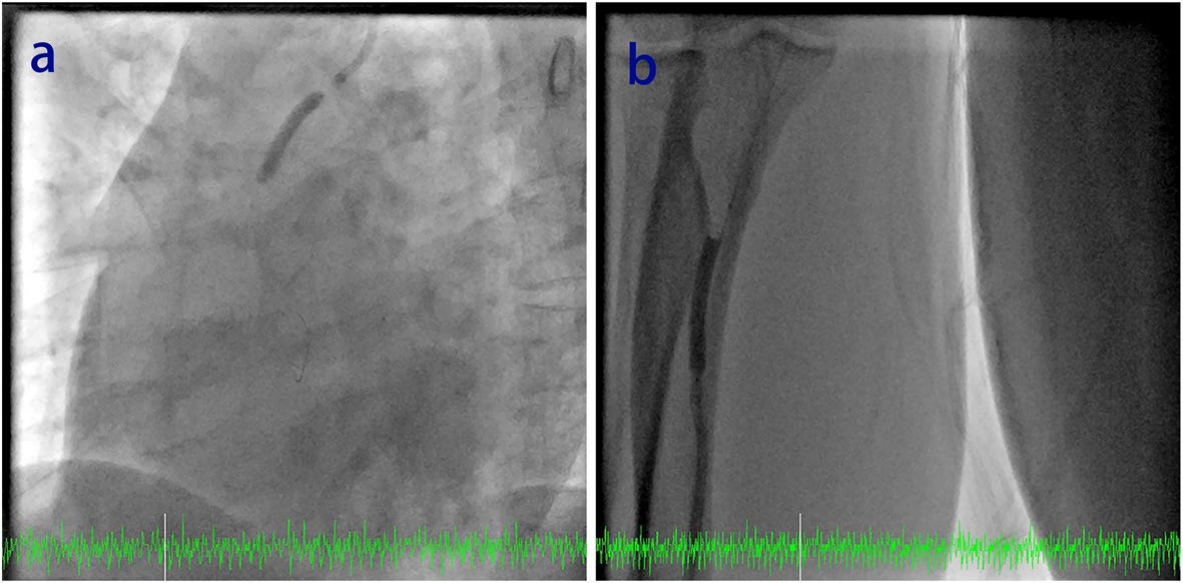
Stent balloon deflation failure and the treatment. **a** The stent balloon was pulled out of the RCA. **b** The 4.0 mm*33 mm stent balloon was pulled to the right radial artery

Under normal conditions, the balloon is inflated to expand the stent, and the stent compresses the atherosclerotic plaque against the vascular wall and restores blood flow to the myocardium. Then, the balloon is withdrawn after deflation. However, after we had dilated the stent balloon with a pressure of 14 atmospheres, the stent balloon could not be deflated. Although the indeflator had achieved negative pressure, the stent balloon still kept expanding and did not deflate. We suspected that the indeflator was broken, so we immediately replaced it with a new one. After the stent balloon was connected, negative pressure was exerted, but the stent balloon still kept expanding. Because the 4.0 mm*33 mm stent balloon continued to block the ostial RCA, resulting in myocardial ischemia, the patient began to complain of general sweating with chest pain, progressing to hypotension and a low heart rate. ECG monitoring in Hemodynamic Recording System showed that the arterial pressure was 63/34 mmHg, and the heart rate was 42 beats/minute, with ST segment elevation in lead II. Immediate action was required, otherwise, the patient's life would be in danger. We contacted the cardiac surgery team by telephone as soon as possible, to activate bystander cardiac surgery. Meanwhile, we forced the stent balloon out of the RCA (Fig. 2a, Supplementary Video 5). When the stent balloon was extracted from the RCA and was in the ascending aorta, the patient's discomfort immediately improved, and blood pressure and heart rate gradually returned to normal.

Another problem was removing the dilated stent balloon from the body. To prevent the balloon from traveling into the cerebral artery or descending aorta, we first pulled the stent balloon to the right radial artery (Fig. 2b, Supplementary Video 6). Then, we tried again to deflate the balloon. Two approaches were considered: one was to dilate the balloon at a pressure greater than the rated burst pressure (RBP). In this way, the volume of the balloon would be reduced after the contrast agent flowed out. Because of the fear of radial artery damage with balloon explosion, we rejected this approach; Another method was to puncture the balloon with a hard guide wire to allow the contrast agent to flow out. However, repeated attempts to puncture the balloon with a Conquest Pro guide wire (Asahi, Tokyo, Japan) failed. Thus, we forced the indeflator with a continuous negative pressure, so that the defect balloon could be retracted into the guiding catheter due to a little partially deflation. Finally, the balloon in the expanded state and the guiding catheter were successfully pulled out of the body.

**Fig. 3:**
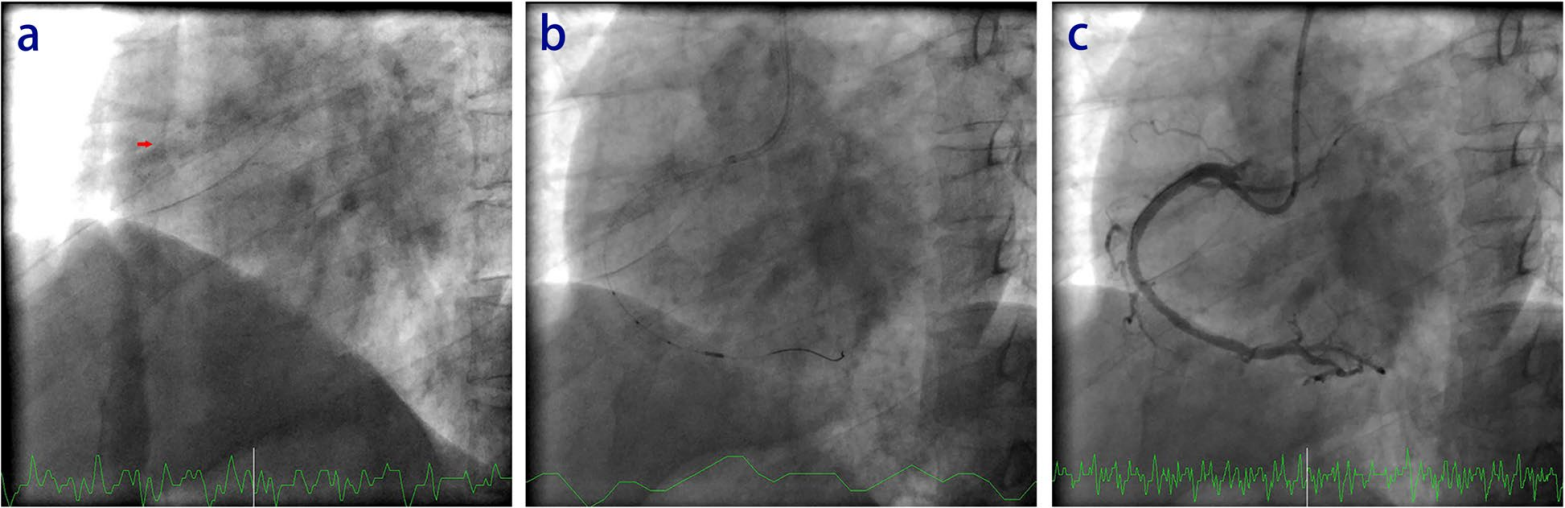
Post-PCI angiography of the RCA with IVUS detection. **a** Radiography of the RCA was performed to confirm that the stent was still inside the proximal RCA. The red arrow points to the stent. **b** Intravascular ultrasound was performed to check the stents in the RCA. **c **Angiography was finally performed to check the RCA

We reviewed the radiography of the RCA and confirmed that the stent was still inside the proximal RCA and had not slipped away (Fig. 3a). Intravascular ultrasound (IVUS) was performed (Fig. 3b, Supplementary Video 7), because we were concerned that the RCA or the stent had been damaged when the stent balloon was forced out of the RCA. Angiography of the RCA was finally performed, and re-examination showed that the stent had expanded completely and was well apposed, with the stenosis of the RCA opened (Fig. 3c, Supplementary Video 8). There was no obvious intimal lesion on either side of the stent. We tested the stent balloon in vitro, and the stent balloon again failed to deflate, confirming that this was not a case of transitory failure of balloon deflation (Supplementary Video 9). After the operation, the patient returned to the ward and was discharged three days later. At the 6-month follow-up, the patient did not have any discomfort.

## Discussion

Deflation failure of a stent balloon is an extremely rare complication of PCI [8]. The deflation failure of a balloon following inflation occurs mostly due to a mechanical obstruction in the shaft of the balloon [9, 10]. These obstructions block the channel, the patency of which is required for the development of negative pressure adequate for balloon deflation. In this case, the stent company inspected the stent balloon after the operation. It was found that the balloon weld, which marks the site where the balloon is laser welded to the shaft, was compromised by a manufacturing defect. Thus, a one-way valve had been formed in the lumen of the balloon. When positive pressure was applied for inflation, the contrast media could pass through, but the negative pressure of deflation would block the lumen. Therefore, balloon deflation failure occurred. According to the evaluation of the stent company, this defect of the balloon was an isolated case, and did not occur again in the following days. Thus, the company did not perform a recall or warning.

There are various stepwise strategies that may be adopted by the operator to approach the dilemma of balloon deflation failure. The first option is the application of negative suction pressure. The second option is to inflate balloon beyond maximal rated pressure to facilitate its bursting. The third option is transection of the balloon shaft. The fourth option involves extracting all systems. The fifth option is to puncture the balloon using the stiff end of a guide wire, either a 0.014″ or 0.018″ guide wire [11, 12]. Of course, the last option is to send the patient for surgical retrieval of the balloon if the other methods proved ineffective [13]. Generally, these options are included in our normal plan for dealing with balloon deflation failure. However, in this case, the patient's hemodynamics were very unstable, and the situation was urgent. We did not have enough time to try the methods above, which were time consuming and would cause some inevitable delay. Therefore, given the emergency, we directly pulled the balloon out of the proximal RCA with gentle force. Indeed, this approach was not always recommended. Especially when an undeflatable balloon were entrapped more distally in the RCA or other coronary arteries, the cardiac surgery should be activated, because more serious risks such as coronary rupture, tamponade, malignant arrhythmias and stent emigration might occur. Fortunately, once the balloon was pulled out of the RCA and into the radial artery, the coronary artery blood flow was immediately restored. Thus, the hemodynamics improved, which kept the patient safe. We then had enough time to try various methods for getting the balloon out of the body. Finally, we were successful, and surgery was avoided.

## Conclusions

Cardiologists engaged in cardiovascular intervention should be prepared for complications such as balloon deflation failure. In patients with hemodynamic stability, the technique of puncturing the balloon with a stiff guide wire may be considered. However, if the patient is unstable, it is urgent to immediately restore blood flow in the blocked coronary artery. After the patient's hemodynamics are stable, the remaining related complications can be managed.

## Supplementary Information

**Additional file 1: Supplementary Video 1.**  A CAG showed 90% stenosis in the proximal RCA and diffuse and severe stenosis in the middle RCA with a balanced coronary dominant circulation.

**Additional file 2: Supplementary Video 2.**  The stenosis of the RCA was dilated with a 2.0 mm*20 mm compliance balloon.

**Additional file 3: Supplementary Video 3. **A 3.0 mm*33 mm DES (Firebird, MicroPort, China) was implanted in the middle RCA.

**Additional file 4: Supplementary Video 4.**  A 4.0 mm*33 mm DES (Firebird, MicroPort, China) was implanted in the proximal RCA.

**Additional file 5: Supplementary Video 5.**  A 4.0 mm*33 mm stent balloon with deflation failure was forced out of the RCA.

**Additional file 6: Supplementary Video 6.**  The 4.0 mm*33 mm stent balloon with deflation failure was pulled into the RCA.

**Additional file 7: Supplementary Video 7.**  IVUS was performed to confirm that the stent remained in the proximal RCA and was not damaged.

**Additional file 8: Supplementary Video 8.**  Angiography of the RCA was finally performed, and re-examination showed that the stent had expanded completely and was well apposed, with the stenosis of the RCA opened.

**Additional file 9: Supplementary Video 9.** The stent balloon also failed to deflate in vitro, confirming that the problem was not a transitory failure to deflate.

## Data Availability

The data analyzed in the case report are not publicly available due to the privacy policy of the hospital, but are available from the corresponding author upon reasonable request.

## Abbreviations

CAD: Coronary artery disease
MI: Myocardial infarction
CAG: Coronary angiography
hsTnI: High-sensitivity troponin I
PCI: Percutaneous coronary intervention
ECG: Electrocardiogram
RCA: Right coronary artery
IVUS: Intravascular ultrasound
DES: Drug-eluting stent
RBP: Rated burst pressure
